# Cytocidal Activities of Topoisomerase 1 Inhibitors and 5-Azacytidine against Pheochromocytoma/Paraganglioma Cells in Primary Human Tumor Cultures and Mouse Cell Lines

**DOI:** 10.1371/journal.pone.0087807

**Published:** 2014-02-07

**Authors:** James F. Powers, Parimal G. Korgaonkar, Stephanie Fliedner, Alessio Giubellino, Karel Pacak, G. Gary. Sahagian, Arthur S. Tischler

**Affiliations:** 1 Department of Pathology, Tufts Medical Center, Boston, Massachusetts, United States of America; 2 Small Animal Imaging/Preclinical Testing Facility, Tufts University School of Medicine, Boston, Massachusetts, United States of America; 3 Program in Reproductive and Adult Endocrinology, Eunice Kennedy Shriver National Institute of Child Health and Human Development, National Institutes of Health, Bethesda, Maryland, United States of America; 4 1^st^ Department of Medicine, University Medical Center Schleswig-Holstein Lübeck, Lübeck, Germany; University of Louisville, United States of America

## Abstract

There is currently no effective treatment for metastatic pheochromocytomas and paragangliomas. A deficiency in current chemotherapy regimens is that the metastases usually grow very slowly. Drugs that target dividing tumor cells have therefore had limited success. To improve treatment, new strategies and valid experimental models are required for pre-clinical testing. However, development of models has itself been hampered by the absence of human pheochromocytoma/paraganglioma cell lines for cultures or xenografts. Topoisomerase 1 (TOP1) inhibitors are drugs that interfere with mechanisms that maintain DNA integrity during transcription in both quiescent and dividing cells. We used primary cultures of representative human tumors to establish the cytotoxicity of camptothecin, a prototypical TOP1 inhibitor, against non-dividing pheochromocytoma/paraganglioma cells, and then employed a mouse pheochromocytoma model (MPC) to show that efficacy of low concentrations of camptothecin and other TOP1 inhibitors is increased by intermittent coadministration of sub-toxic concentrations of 5-azacytidine, a DNA methylation inhibitor that modulates transcription. We then tested the same drugs against a clonal MPC derivative that expresses CMV reporter-driven luciferase and GFP, intended for in vivo drug testing. Unexpectedly, luciferase expression, bioluminescence and GFP expression were paradoxically increased by both camptothecin and SN38, the active metabolite of irinotecan, thereby masking cell death. Expression of chromogranin A, a marker for neuroendocrine secretory granules, was not increased, indicating that the drug effects on levels of luciferase and GFP are specific to the GFP-luciferase construct rather than generalized cellular responses. Our findings provide proof of principle for use of TOP1 inhibitors against pheochromocytoma/paraganglioma and suggest novel strategies for enhancing efficacy and reducing toxicity by optimizing the combination and timing of their use in conjunction with other drugs. The paradoxical effects of TOP1 inhibitors on luciferase and GFP dictate a need for caution in the use of CMV promoter-regulated constructs for cancer-related imaging studies.

## Introduction

Pheochromocytomas (PCC) are neuroendocrine tumors that arise from chromaffin cells in the adrenal medulla. Closely related extra-adrenal tumors are arbitrarily classified by the World Health Organization as paragangliomas (PGL) [1]. Up to 30% of PCC/PGL give rise to metastases, for which there is currently no effective treatment [2]. An additional subset of these tumors is surgically unresectable. A major deficiency in current treatment strategies that they do not account for the fact that, in contrast to many other types of malignant tumors, PCC/PGL usually grow very slowly and most of the cells are quiescent at any given time. Mitotic counts and expression of cell cycle markers both in primary tumors and in their metastases are usually very low [3]. Treatments that target replicating tumor cells or tumor angiogenesis have therefore met with only limited success. Patients with metastases or inoperable tumors often die from complications of catecholamine hypersecretion, or from invasive and expansile tumor growth that occurs over many years.

The need to improve treatment of metastatic or unresectable PCC/PGL requires new strategies and a valid experimental model for pre-clinical testing of those strategies. However, development of a model has itself been hampered by failure to establish any human PCC cell lines for cell culture or xenograft studies, despite many efforts to establish them over a period of more than 35 years [4] and several initially promising reports. Factors contributing to these failures are that there are very few dividing cells even in vivo, as shown by staining for Ki-67 or other markers [3]., and that whatever dividing cells are present immediately undergo growth arrest in culture [4]. One recent paper reports the establishment of a putative PCC progenitor line using a TERT construct [5], but the cells appear to bear minimal resemblance to PCC and are also not generally available.

Topoisomerases are enzymes that alleviate topological stresses such as supercoiling that occur when DNA strands are unwound during transcription or replication. The enzymes function by introducing transient single strand (topoisomerase 1, TOP1) or double strand (topoisomerase 2, TOP2) DNA breaks. Inhibition of topoisomerases initiates apoptotic cell death [6], [7]. The prototypical TOP1 inhibitor, camptothecin, causes DNA damage both during S-phase and during transcription [8], thereby potentially activating apoptotic pathways in both dividing and non-dividing cells. Further, cytotoxicity of camptothecin on both dividing and non-dividing PCC cells was demonstrated by Greene and colleagues, who first showed in the 1990’s that the drug causes apoptotic death of nerve growth factor-treated PC12 cells [9]. We therefore hypothesized that camptothecin and other TOP inhibitors might be effective chemotherapeutic agents for treatment of metastatic PCC/PGL.

Camptothecin is known to be toxic to many kinds of cancer cells, but systemic toxicity and a long time course required for its effect have prevented its general use in chemotherapy. Several camptothecin analogs currently are in use, including topotecan and irinotecan. These have been employed in combination with other agents to treat a variety of aggressive neuroendocrine carcinomas, with mostly modest results in terms of patients’ survival [10], [11]. However, new TOP1 and TOP2 inhibitors are under development [6], [7], as is a particle-bound form of camptothecin that might have reduced toxicity and increased efficacy [12], [13], and increasing numbers of publications in recent years attest to growing awareness of the potential value of camptothecin or its analogs as chemotherapeutic agents.

This *in vitro* study was undertaken in preparation for the clinical availability of new camptothecin derivatives, and had two objectives. The first was to test the effectiveness of TOP1 inhibition against pheochromocytoma cells using camptothecin to obtain proof of principle. The second. was to develop strategies for enhancing the efficacy and reducing the toxicity of TOP1 inhibitors by optimizing the combination and timing of their use in conjunction with other drugs. Because there are no human PCC/PGL cell lines, we first used primary cultures of representative human tumors to establish the cytotoxicity of camptothecin against non-replicating human PCC/PGL cells. We then used a mouse pheochromocytoma cell line (MPC) as a model to further test camptothecin and other TOP1 inhibitors in conjunction other drugs. As a prototype for complementary drugs, we used the DNA methyltransferase inhibitor, 5-azacytidine (5-aza), which we hypothesized would potentiate the effect of camptothecin because it is known to increase transcription of multiple genes by causing promoter demethylation. Combinations of existing TOP1 inhibitors with other drugs have previously been tested against other tumors in clinical or experimental settings, with mixed results. These include a strategy for staggered initiation of combined treatment with irinotecan and 5-aza-2′-deoxycytidine reported by Ishiguro et al to be highly effective against a colorectal cancer cell line *in vivo* and in cell culture [14]. Promoter demethylation in response to 5-aza has until recently been considered to be dependent on DNA replication in dividing cell populations. However, increasing evidence indicates that DNA can be demethylated in a process of dynamic remodeling that occurs in both dividing and non-dividing cells [15].

## Materials and Methods

### Ethics Statement

Studies of human tumor samples were approved by the Institutional Review Boards of the National Institutes of Health and Tufts Medical Center. Patients provided written informed consent.

### Human Tumor Cultures

Seven human PCCs/PGLs representing different genotypes and locations were enzymatically dissociated and plated in 35 mm culture dishes at a density of ∼5000 cells/dish in RPMI 1640 medium with 15% fetal bovine serum. Cultures were maintained for 1–2 weeks before the start of drug testing to allow for firm attachment. During the pre-testing interval, representative dishes were pulsed for at least 5 days with 10 uM bromodeoxyuridine (BrdU), which is incorporated into the DNA of proliferating cells, then fixed and double stained for BrdU and tyrosine hydroxylase (TH), a marker of catecholamine-synthesizing ability, to discriminate tumor cells from non-neoplastic fibroblasts and other cell types in primary cultures [16]. At the start of drug testing, camptothecin (Sigma Chemical Co, St Louis, MO) and/or additional drugs were added for the time intervals and at the concentrations indicated in the figure legends. At the end of the experiments cultures were fixed and stained for TH in order to identify surviving tumor cells.

To measure drug-induced cytotoxicity, surviving TH-positive cells were counted in an area of the culture dish defined by a randomly placed 22×22 mm square coverslip.

Protocols for dissociation and culture of PCC/PGL cell cultures were as previously described for similar studies by Powers et al [17]. Cytotoxicity assays were performed without knowledge of tumor genotype or location until final tabulation of the data.

### Mouse Pheochromocytoma Cell Lines

The mouse pheochromocytoma cell line MPC 4/30PRR was developed in our laboratory [18] and previously utilized for testing of other potential chemotherapeutic agents [19]. Cells tested were from passages ∼20–25 maintained as described by Powers et al [18]. The less differentiated derivative of MPC 4/30PRR designated MTT (for mouse tumor tissue) established from MPC tumor tissue formed after reinjection of the original cell line into nude mice [20], was maintained as described by Martiniova et al [20]. The MPC and MTT lines are complementary for drug testing purposes in that MTT best reflects aggressive metastases, while MPC is better differentiated and more comparable to slowly growing, hormonally active metastases [21] [22].

We derived an additional cell line designated MPC 4/30/PRR GL-9 (abbreviated to MPC GL-9) expressing copepod green fluorescent protein (copGFP) and firefly luciferase from MPC 4/30PRR by transducing the cells with a pre-packaged lentiviral construct (GreenFire1, SBI Systems) containing both genes under control of the CMV promoter. Infection was performed according to the manufacturer’s protocol and MPC GL-9, which stably expresses high levels of luciferase, was cloned from a single transduced cell identified by its GFP fluorescence. Aside from expression of its two marker proteins, MPC G-L9 is similar to its parent tumor. It is intended to be used for in vivo bioluminescence imaging of tumor deposits comparably to the recently described MTT derivative known as MTT-luc [23].

### Cytotoxicity Testing and Immunostaining of MPC and MTT Cell Lines

Drug testing regimens were as described in the figure legends. Cytotoxicity against MPC 4/30PRR and MTT cells was tested in parallel using the XTT colorimetric assay to quantitate cell survival (Cell Proliferation Kit II, Roche, Indianapolis IN). Additional XTT assays were performed to compare cytotoxicity against MPC 4/30PRR and MPC GL-9 cells, and a parallel assay using bioluminescence as the reporter was performed on MPC GL-9 cells. The luminescence assay was essentially as described by Giubellino et al [23]. All cytotoxicity experiments with mouse cell lines were performed on 3 occasions unless otherwise specified.

To assess BrdU incorporation into MPC cells, cultures were pulsed with 10 uM BrdU for 24 h, then fixed and stained for BrdU and TH by the same method as the primary human cell cultures.

### Effects of Other TOP1 Inhibitors on MPC and Human Pheochromocytoma Cells

In order to determine the cytotoxicity of camptothecin analogs currently in clinical use, we first used MPC in XTT assays to test four drugs: camptothecin, topotecan, irinotecan and SN38, the active metabolite of irinotecan. The concentrations of these drugs were based on published preclinical studies of other cell types [24].

### Assessment of Apoptosis

To test and compare the effects of different combinations of camptothecin and 5-aza on apoptosis, MPC cells were cultured with the two drugs separately or in combination for up to 2 weeks, with a switch in sequence of addition at one week corresponding to the schedule of cytotoxicity testing by XTT assay. Immunoblots were then probed for a 25 kDa fragment of poly (ADP-ribose) polymerase (PARP), that is cleaved from the 116 kDa nuclear enzyme by activated caspase 3 and serves as an apoptosis marker [25]. To confirm the morphological changes of apoptosis, fixed cultures were stained with 4′-6-diamidino-2-phenylindole (DAPI, 0.5 uM) (Abbot Molecular, Abbott Park IL) and examined by fluorescence microscopy.

### Immunoblots

Protocols for protein extraction and immunoblotting were as previously described [17]. Cleaved PARP was detected with a rabbit monoclonal antibody from Epitomics Inc, (Burlingame, CA, USA). Firefly luciferase protein was detected with mouse monoclonal antibody Luci 1–107 from Abcam (ab7358, 1∶000), copepod GFP was detected with TurboGFP polyclonal rabbit antibody PA5-22688 from Thermo Scientific Pierce, and chromogranin A was detected with a polyclonal rabbit antibody provided by Dr. Reiner Fischer-Colbrie, Innsbruk, Austria.

### Statistics

Statistical significance of drug effects on survival of human PCC/PGL cells studied by immunocytochemistry on MPC cells in XTT assays was analyzed by one-way ANOVA. Statistics for luminescence imaging analyses are as described by Tao et al [26].

## Results

### Cell Culture Studies

#### Primary human tumor cell cultures

**Figure 1:**
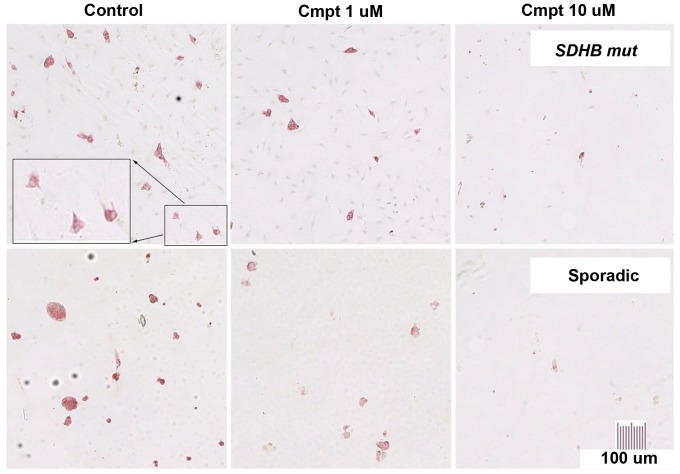
Killing of cells from *SDHB*-mutated and apparently sporadic human PCC/PGL s by camptothecin. Dissociated human tumor cells in primary cultures maintained with 0 (control), 1 or 10 uM camptothecin (Cmpt) for 2 wks, then fixed and stained for TH (red cytoplasm) to discriminate the tumor cells from other cell types. At 10 uM, camptothecin eliminated almost all background cells (faintly visible as hematoxylin-counterstained blue nuclei in top row control and 1 uM panels) and was therefore considered too toxic for the purposes of this investigation.

Progressive, dose-dependent killing of human PCC/PGL cells was observed in cultures maintained for up to 2 weeks in the presence of camptothecin *versus* control medium. At two weeks, mean survival was approximately 13% with 10 uM camptothecin and 38% with 1 uM (Table 1 and Fig. 1). Cytotoxicity was independent of tumor genotype or location in this series. Staining of additional control cultures for BrdU and TH showed no BrdU incorporation into TH-positive cells and robust incorporation into TH-negative cells in the pre-testing period. This finding was consistent with our previous observations that human PCC/PGL do not proliferate in primary cultures [4], and indicated that the effect of camptothecin on human PCC/PGL cells does not require DNA replication.

**Table 1:** Cytotoxicity of camptothecin against human PCC/PGL cells in primary cultures.

		Surviving Cells/Dish	
		(% of Control)	
		1.0 uM Cmpt	10 uM Cmpt
Tumor	Genotype		
1 PCC	VHL	39.1	18.6
2 PCC	Sporadic-Neg	63.3	29.1
3 PCC	Sporadic -Neg	18.6	4.7
4 PCC	Unknown NT	45.7	
5 PGL	SDHB	33.1	2.2
6 PGL	SDHB	71.7	
7** PGL	SDHB	24.0**	10.1

Dissociated primary tumor cells from PCCs or PGLs representing different genotypes were cultured in the presence of 1 uM or 10 uM camptothecin compared to control medium. Counts were derived by counting all stained cells defined by a randomly placed square coverslip in a 35 mm culture dish (see Figure 1). All counts were done at 2 weeks except for tumor 7 (**), which was counted at 1 week because of extensive cell death caused by particular sensitivity to camptothecin. The two tumors listed as sporadic negative were tested negative for MEN2 *RET* mutation and for *SDHB*, *SDHC* and *SDHD* mutations and deletions.

The proportion of TH-negative contaminating cell types in control cultures was estimated at <10% to >80%, reflecting the composition and varying ease of dissociation of individual tumors. Cytotoxicity of camptothecin against TH-positive tumor cells was not obviously affected by the relative presence or absence of other cell types. However, bystander toxicity on TH-negative cells was evident, particularly in cultures with 10 uM camptothecin, indicating a need for strategies to reduce the effective camptothecin dose.

### MPC versus MTT Cells

**Figure 2:**
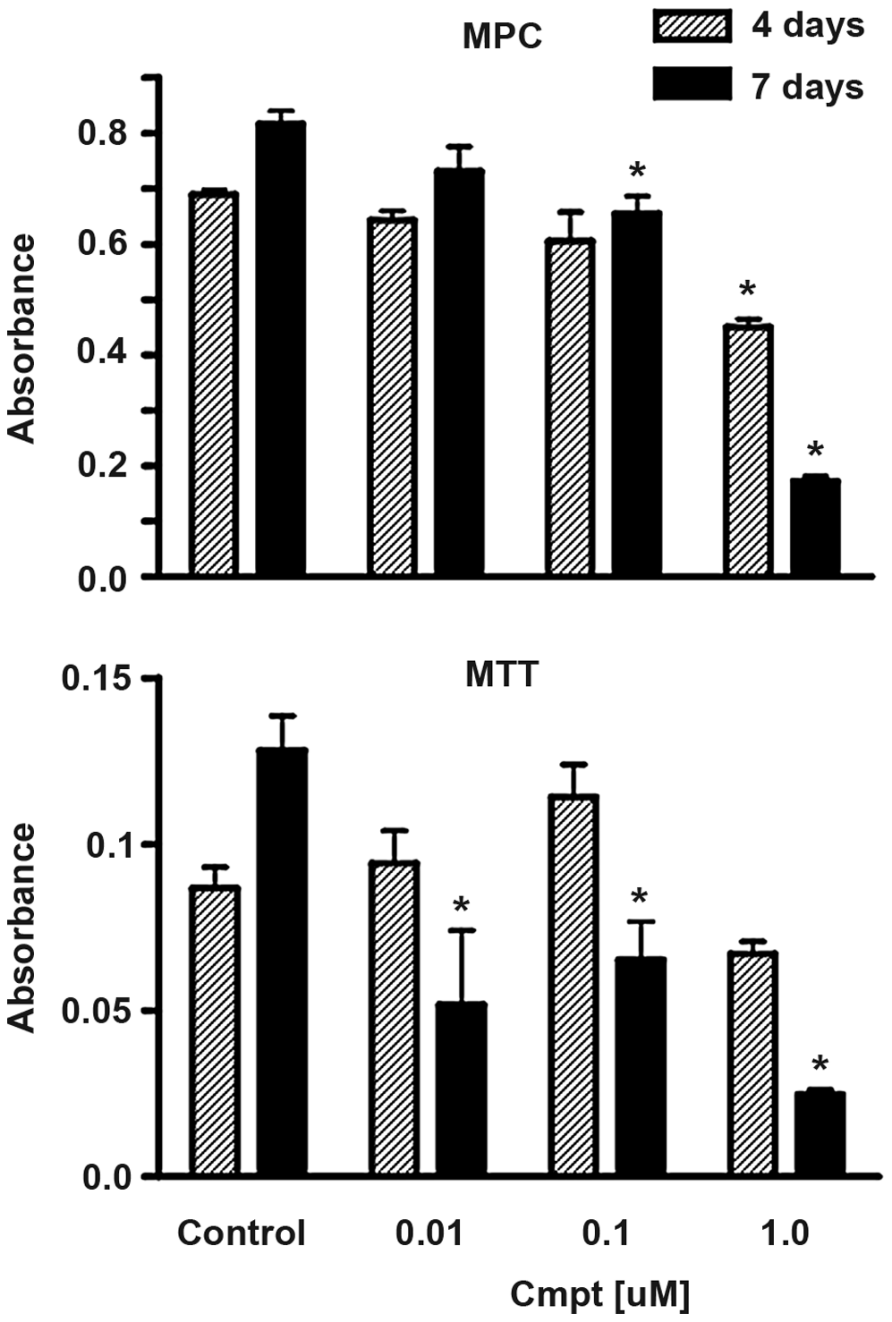
Comparative cytotoxicity of camptothecin against MPC and MTT cells. Parallel tests of camptothecin toxicity on MPC and MTT cell lines at two time points, demonstrate greater sensitivity of the more aggressive MTT line to low camptothecin concentrations. Data are from a representative experiment that was repeated on 2 independent occasions. Bars indicate mean +/− SEM of quadruplicate wells.

Initial comparisons of the MPC and MTT cell lines showed that both lines were more sensitive to camptothecin than their human counterparts, with MTT showing a lower threshold of response than MPC (Fig. 2). Both cell lines showed approximately 20% survival in the presence of 1 uM camptothecin at 7 days and no survival at 7 days in the presence of 10 uM camptothecin. Because the responsiveness of MPC more closely resembled that of human pheochromocytomas, MPC was used as the focus for subsequent studies.

### Responses to Camptothecin and 5-azacytidine

**Figure 3:**
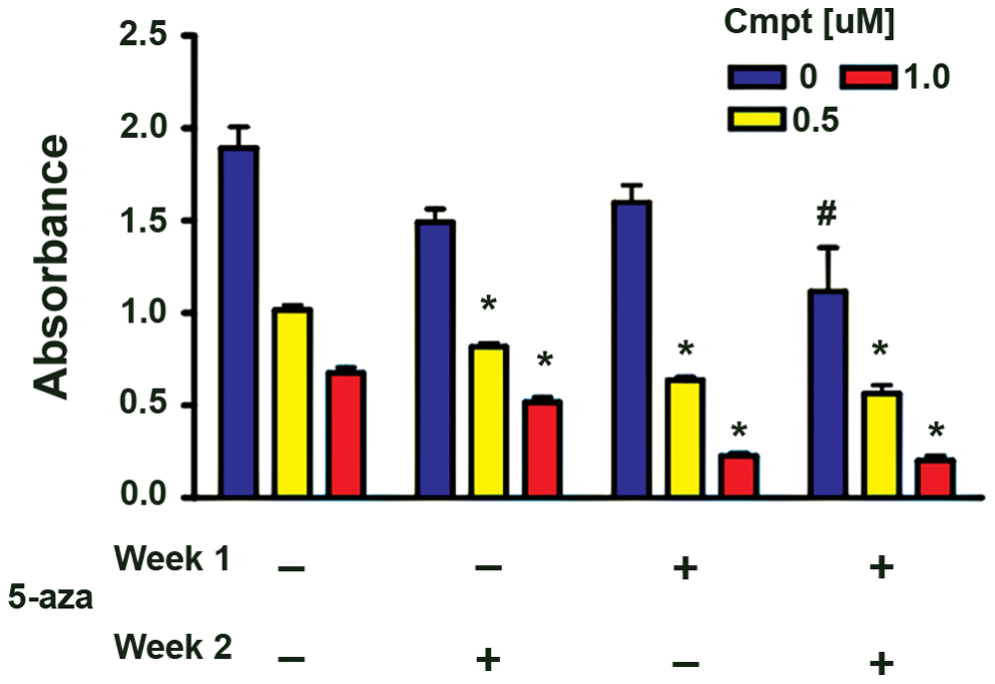
Cytotoxicity of camptothecin against MPC cells is increased in the presence of 5-azacytidine. Cytotoxicity of camptothecin against MPC 4/30/PRR cells was tested in the presence or absence of 5-azacytidine (1 uM) by XTT assay. Absorbance is proportional to cell survival. Captions under each bar indicate whether 5-aza was present during the first week/second week of a 2-week experiment. Data are from a representative experiment that was repeated on 3 independent occasions. Bars indicate mean +/− SEM of quadruplicate wells.

To test the interaction of camptothecin with 5-aza, cytotoxicity assays with MPC cells were performed over a 2-week period with camptothecin present continuously and 5-aza added during either the first or second week (Fig. 3), Two concentrations of camptothecin (0.5 uM and 1 uM) and a single (1 uM) concentration of 5-aza were tested. Mean survival at two weeks was significantly decreased in cultures treated with 5-aza plus camptothecin compared to either concentration of camptothecin alone, However, cooperativity was optimal during the first week of culture and was reduced when 5-aza was present only for the second week. Further, when 5-aza was removed after one week from cultures initially receiving camptothecin plus 5-aza, survival was equivalent to that in cultures containing both drugs for the entire 2 weeks (Fig. 3). A small decrease in survival seen with 5-aza alone was cumulative over the two week period and significant at 2 weeks (Fig. 3). In contrast to human primary cultures, MPC cells from passage numbers used in this study showed approximately 30% BrdU labeling/24 hrs at the onset of cytotoxicity testing (not shown).

**Figure 4:**
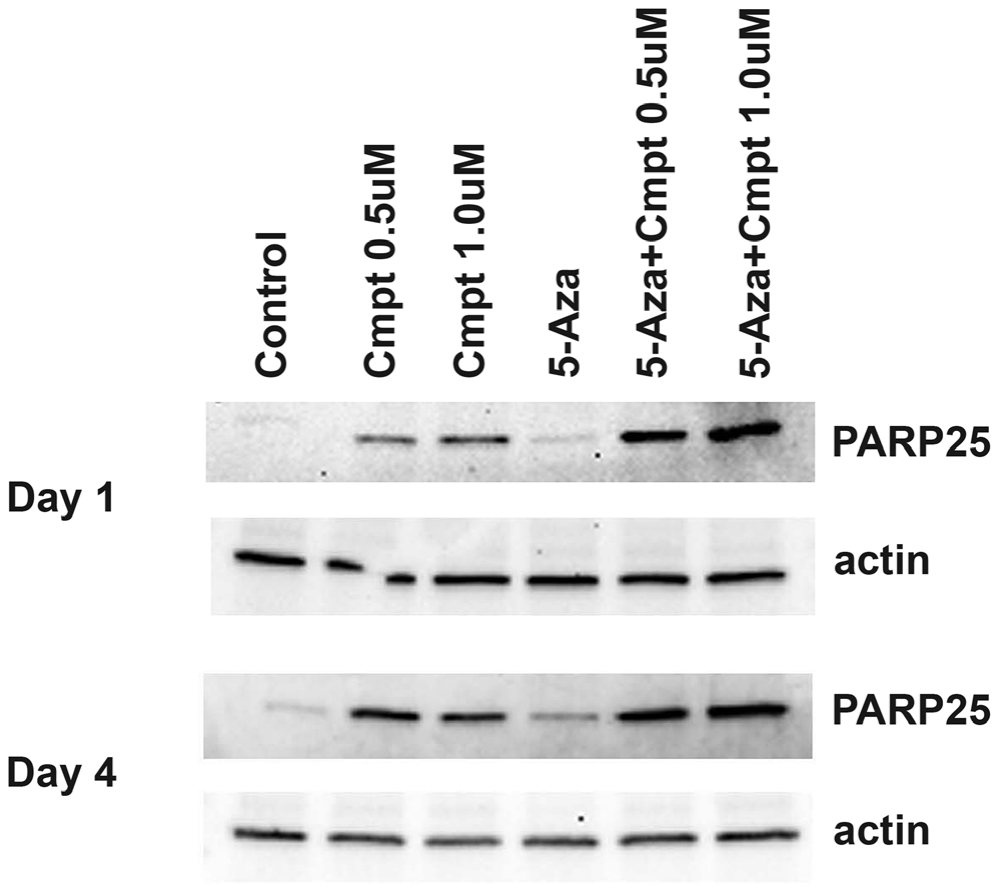
Camptothecin and 5-azacytidine cooperatively increase MPC cell apoptosis. Immunoblots show the cooperative effects of camptothecin and 5-azacytidine on MPC cell apoptosis, which is indicated by the presence of a 25 kDa fragment of PARP. A marked increase in intensity of the PARP25 band is seen at 24 hrs with the combination of camptothecin and 5-aza, with little effect of 5-aza alone. This pattern is still evident, but diminished, after 4 days.

**Figure 5:**
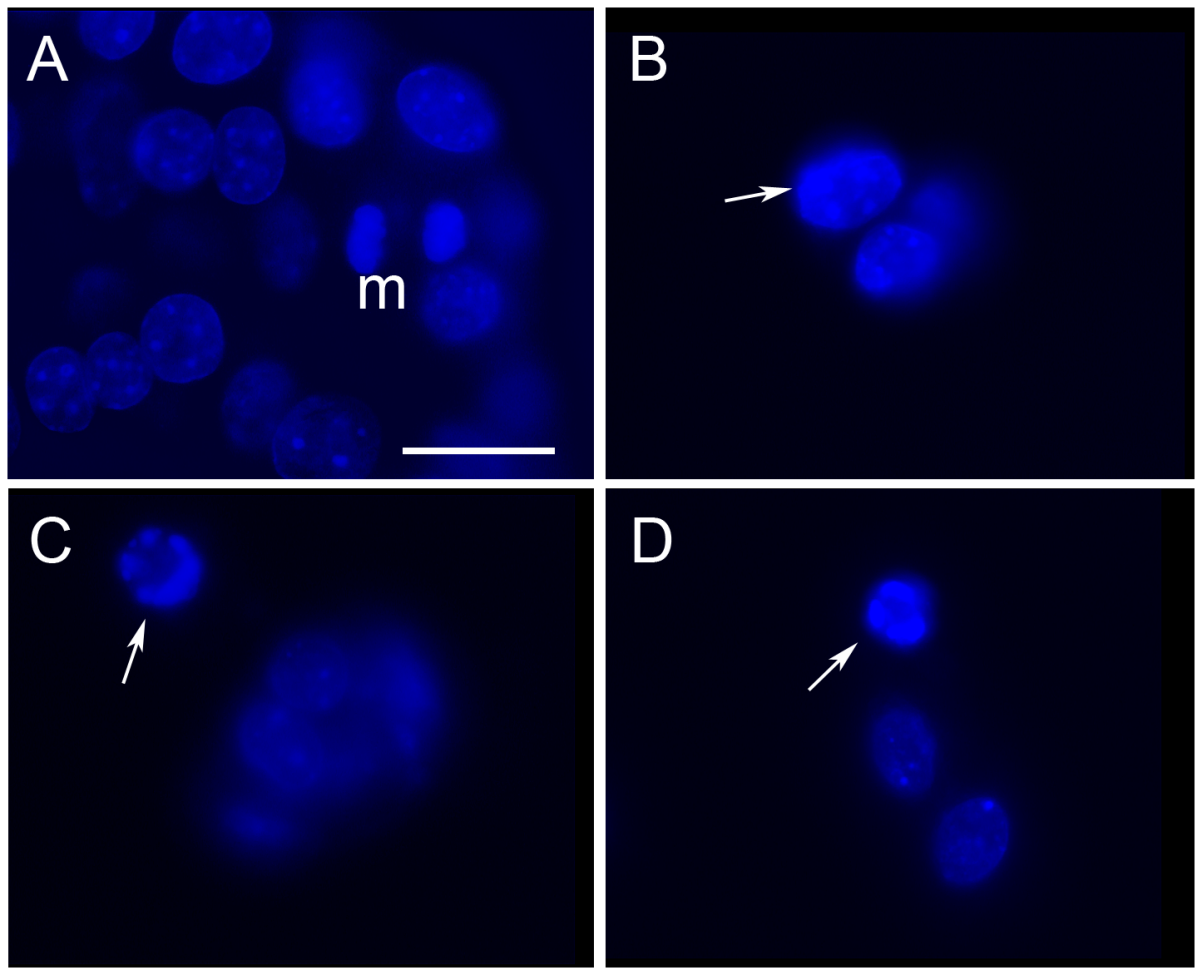
MPC cells treated with camptothecin show morphological changes of apoptosis. Representative fluorescence micrographs showing nuclear morphology of DAPI-stained MPC cultures. Panel A shows nuclei of cells maintained in control medium for 7 days. Nuclei are round to oval with finely stippled chromatin. One mitosis is evident (m). Panels B–D show typical apoptotic changes seen at day 7 in cultures with camptothecin or camptothecin +5-aza. (B, early peripheral margination of chromatin; C, nuclear shrinkage and marked chromatin margination; d, nuclear fragmentation). In addition, B–D contain fewer cells, consistent with ongoing attrition. Bar = 20 um. Original magnification 100 x.

Immunoblots for the cleaved p25 PARP fragment showed a marked increase within 24 hrs in apoptosis caused by the combination of camptothecin and 5-aza, with little or no effect of 5-aza alone (Fig. 4). This pattern of cooperativity was still evident after 4 days (Fig. 4). However, it was no longer detectable at day 7, when the intensity of the PARP band was increased in cultures with 5-aza alone. Consistent with this finding, fluorescence microscopy at day 7 showed many cells with nuclei in final stages of apoptotic death [27] in cultures with camptothecin alone or camptothecin plus 5-aza, and a few similar cells were seen in cultures with 5-aza alone. (Fig. 5).

**Figure 6:**
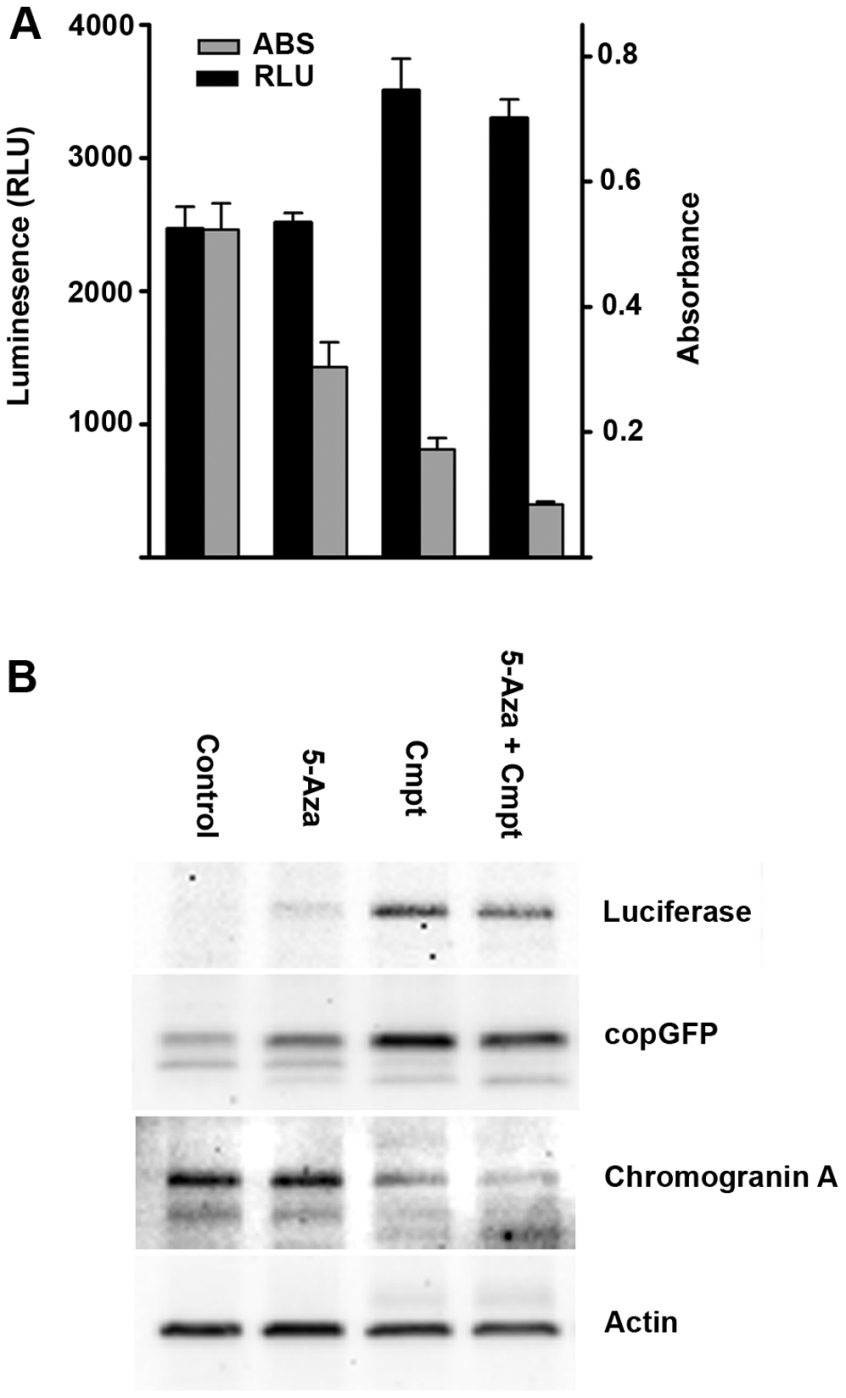
Camptothecin paradoxically increases bioluminescence and luciferase expression. (A) Effects of camptothecin and 5-azacytidine on bioluminescence of MPC GL-9 cells compared to survival measured by Absorbance in XTT assay at 1 week. (B) corresponding immunoblot from the same experiment showing increased levels of firefly luciferase protein and copepod GFP (copGFP) in camptothecin-treated cultures. Expression of CgA is not increased, indicating that the effect is specific for the luciferase construct. The paradoxically increased bioluminescence of cells treated with camptothecin obscured obvious actual toxicity that was quantifiable by XTT assay.

With MPC GL-9, the results of cytotoxicity testing by XTT assay were comparable to those with the parent tumor. However, in the parallel bioluminescence assays a paradoxical increase in luminescence above control levels was caused by camptothecin despite the presence of drug-induced apoptosis and cell death. A comparably large increase was not detectable in the presence of 5-aza alone (Fig. 6A), However, cultures with 5-aza alone maintained a constant level of luminescence in the presence of decreased cell numbers shown by XTT assay, consistent with a smaller luminescence increase. Immunoblotting for luciferase protein supported this interpretation, showing increased band intensities in camptothecin-treated cells and also a small increase in cells treated with 5-aza (Fig. 6B). Parallel increases were seen in GFP bands, while chromogranin A bands in the same immunoblot decreased in response to camptothecin and showed no effect of 5-aza. Because CgA is a marker for neuroendocrine secretory granules, this finding indicates that the drug effects on levels of luciferase and GFP are specific to the GFP-luciferase construct rather than increased granule content or other generalized cellular responses.

### Responses to other TOP1 Inhibitors

**Figure 7:**
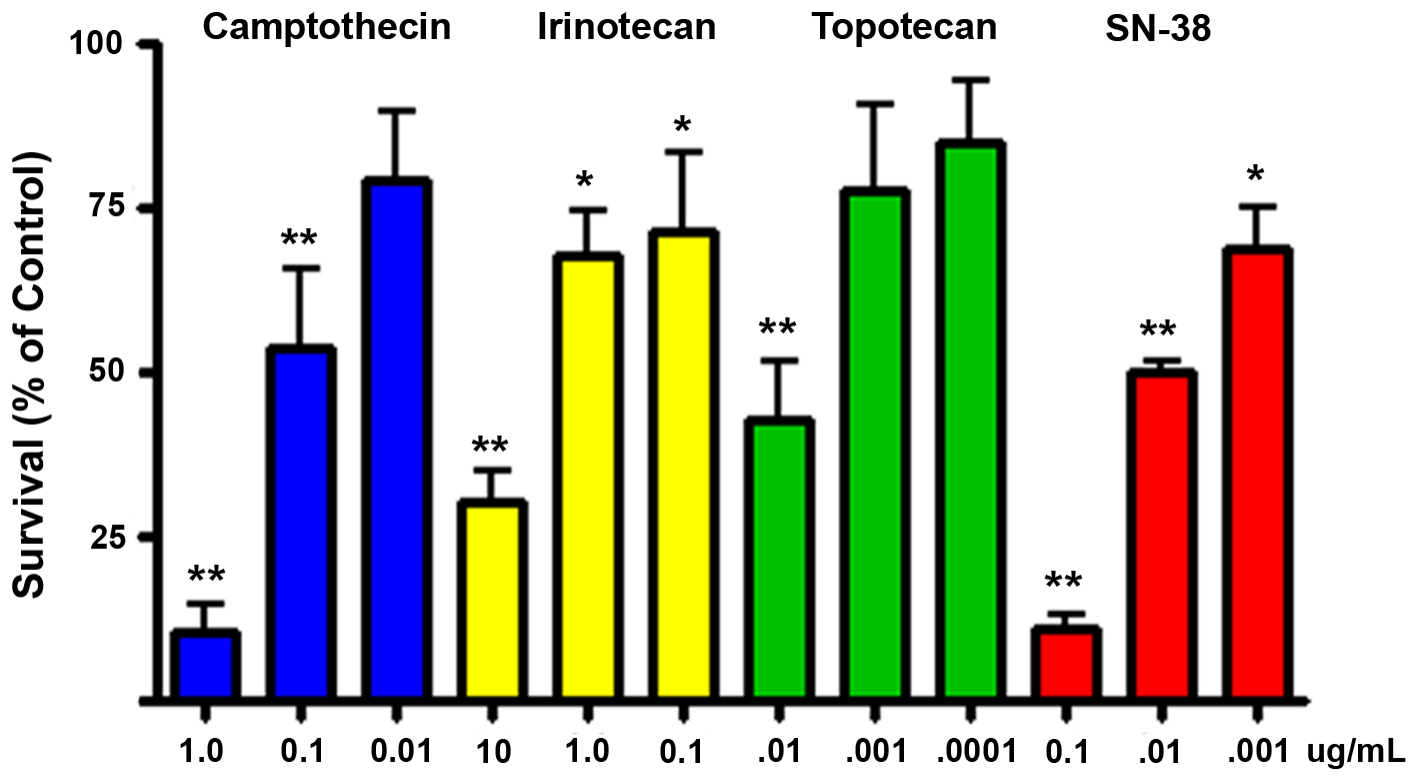
Clinically utilized TOP1 inhibitors show variable toxicities to MPC cells. Concurrent tests of camptothecin versus clinically utilized TOP1 inhibitors on MPC cells in monolayer cultures at one week. Equivalency of camptothecin and SN-38 is seen at 10-fold lower concentrations of SN38. Data are from three independent experiments, each with triplicate wells. Bars indicate mean +/− SEM. (**, p<.01; *, p<.05).

Because native camptothecin is considered too toxic for clinical use, we tested three additional TOP1 inhibitors; topotecan, irinotecan and SN38, the active metabolite of irinotecan, against MPC cells for up to 2 weeks using the same methods as for camptothecin. The concentration ranges tested (0.1–10 ng/mL for topotecan, 1–100 ng/mL for SN38, 0.1–10 ug/mL for irinotecan) were chosen to match published *in vitro* tests of these drugs against other tumors [24]. The relatively high concentration of irinotecan required in cell cultures reflects the fact that the enzyme required for *in vivo* metabolic activation is not present. On a molar basis the most potent drug was SN38, which was approximately 10 times as potent as camptothecin, with 100 ng/mL SN38 (0.26 uM) or 1 ug/mL camptothecin (2.7 uM) each causing ∼90% cell death (Fig. 7). Irinotecan was less potent as expected but did show some effect, suggesting that some conversion to SN38 might take place in the cultures.

**Figure 8:**
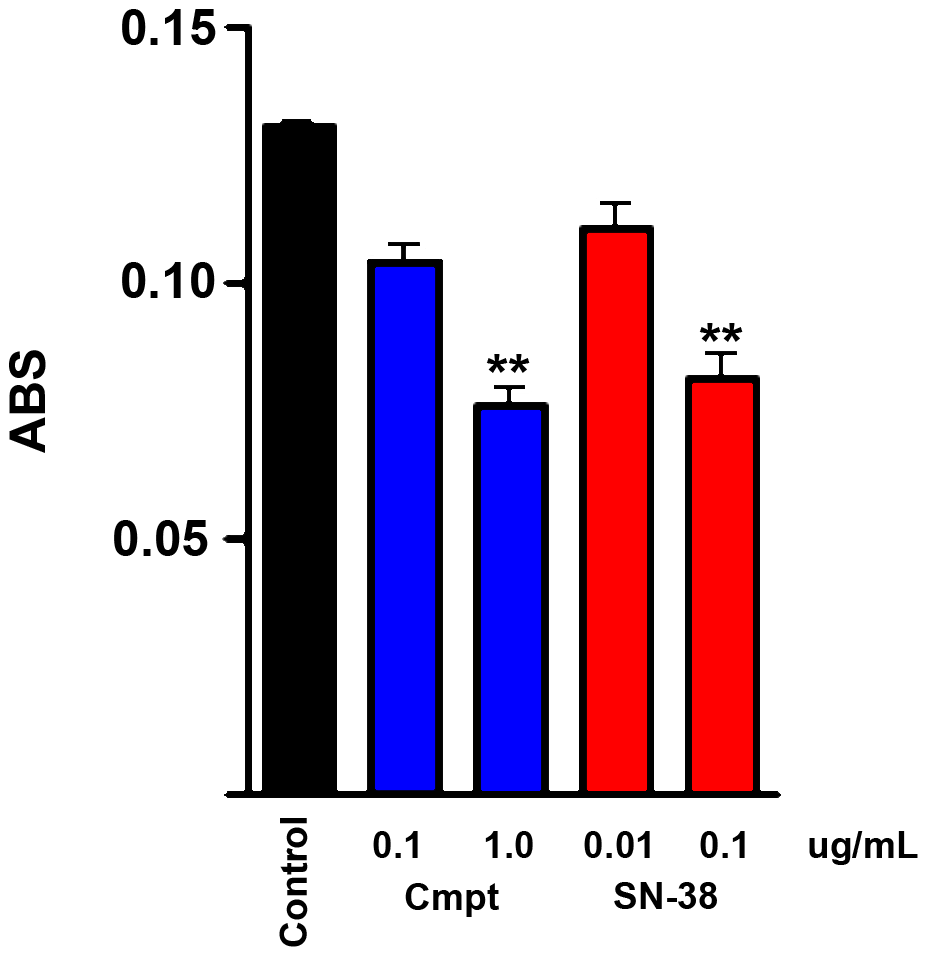
The active metabolite of irinotecan is toxic to primary human pheochromocytoma cells. XTT assay results showing killing of human PCC cells by camptothecin and SN-38. Cultures were treated with the indicated drug concentrations for one week. Data represent mean +/− SEM of triplicate wells. (**, p<.01).

Following the above result, we tested SN38 in a XTT assay against one representative human pheochromocytoma for which a sufficient number of highly purified tumor cells could be obtained by multiple rounds of plating and differential detachment prior to testing. The human cell population tested in the XTT assay was confirmed to consist of >90% TH-positive cells by immunohistochemical staining of an additional culture. As shown in Fig. 8, SN38 killed human PCC cells similarly to camptothecin, although both drugs were less effective against human PCC than against MPC cells. A set of immunohistochemically stained cultures of the same tumor tested for 2 weeks with SN-38 as described for the camptothecin experiments in Fig. 1 showed 38.6% survival. This experiment also included 5-aza, which showed no enhancement of the SN-38 effect (37.3% survival).

## Discussion

In this study we used primary cultures to show that human PCC/PGL are highly sensitive to camptothecin, a prototypical TOP1 inhibitor. Importantly, the representative tumors tested included three from patients with germline *SDHB* mutations, which are the most likely to metastasize [2]. All *SDHB*-mutated tumors were sensitive to camptothecin, and one such tumor was extremely sensitive, supporting a potential role for TOP1 inhibitors in treating metastases. We then demonstrated that mouse pheochromocytoma cells respond similarly, providing a physiologically relevant model for further studies. Other authors have previously advocated the use of primary cultures or primary tumor xenografts for testing chemotherapy regimens in parallel with human tumor cell lines because it is recognized that even human cell lines often do not accurately reflect the properties of their parent tumors [24]. The use of mouse cells was necessary in this study because there are currently no human PCC/PGL cell lines. However, we were unable to fully rely on primary cultures because of the rarity of PCC/PGL and the considerable difficulty of obtaining cells in sufficient number and purity from any individual tumor. It was therefore important to document that mouse pheochromocytoma cells represent in fact a valid model.

A major consideration for testing any drug on human PCC/PGL is the fact that most human PCC/PGL *in vivo* grow very slowly even when metastatic, and the vast majority of cells are non-replicating *in vivo* as well as in cell culture [3]. In this study we therefore first used human primary cultures to provide the foundation for further testing by establishing the fact that camptothecin is cytotoxic to non-replicating PCC/PGL cells. We then employed the mouse model to show that efficacy of a low concentration of camptothecin is increased by intermittent coadministration of a minimally toxic concentration of 5-aza, and that efficacy of the drugs in combination can be optimized by timing the sequence and duration of 5-aza administration. In the MPC model, the optimal effectiveness of the latter strategy was seen in a period of less than one week.

The rationale for use of 5-aza was our hypothesis that it would sensitize cells to camptothecin by its reported ability to alter transcription in both replicating and non-replicating cells [28], facilitated by dynamic DNA remodeling. [15]. However, we were unable to detect an effect of 5-aza in studies of representative non-replicating human PCC cells tested similarly to MPC. It remains possible that other relatively non-toxic drugs known to evoke large transcriptional changes in pheochromocytoma cells and in non-dividing neurons might be similarly tested. These include caffeine [29] and lithium [30]. In addition, methylation inhibitors might still play a role in the treatment of metastatic PCC/PGL because the growth of metastases is dependent on the small numbers of dividing cells that these deposits do contain. Those dividing cells are likely to be particularly susceptible to methylation inhibitors because the tumors are often characterized by a “methylator phenotype” [31], [32].

Modes of action of 5-aza in addition to effects on DNA methylation have not been ruled out in MPC cells, and methylation-independent effects of 5-aza or (DAC) on transcription have been reported in other cell types [33]. However, at the concentration tested, the relatively potent effect of 5-aza in conjunction with TOP1 inhibition versus the small effect alone at least suggest that the proapoptotic effect of the drug is strongly, if not completely, transcription dependent.

Although generally concordant effects of camptothecin in the primary human and MPC models validates the use of MPC cells to study the cytotoxicity of TOP1 inhibitors, a caveat is that the effects of low camptothecin concentrations were greater on MPC cells than on their human counterparts. This is very likely attributable to the fact that MPC cells do proliferate, increasing their sensitivity to some chemotherapy drugs. In contrast to human PCC/PGL, which showed no BrdU incorporation at the onset of cytotoxicity testing, 30% of MPC cells in this study showed BrdU labeling in 24 hours. Further, in preliminary studies we found that exposure to 5 aza at a relatively high concentration (50 uM) for 72 hours can completely and reversibly inhibit BrdU incorporation (JF Powers and AS Tischler, unpublished data). In the present studies, inhibition of proliferation could have contributed only minimally to the cooperativity between camptothecin and the low concentration of 5-aza that we employed because a robust increase in apoptosis assayed by PARP cleavage was observed in response to the two agents within 24 hours, and markedly decreased cell numbers were detected by the XTT colorimetric assay within one week. In contrast, significantly decreased cell number in cultures with 5-aza alone were detectable by XTT assay only after 1–2 weeks. Nonetheless, the inhibitory effect of a high concentration of 5-aza on MPC cell proliferation suggests a further novel strategy that could be developed in future studies. Specifically, the effect could be exploited by cyclic timing and staggering of drug administration, so that TOP inhibitors first target cells in which genes are activated during 5-aza -induced cytostasis, and additional cells are then targeted when 5-aza is removed to permit reactivation of genes controlling cell cycle progression. That approach might be applicable to treatment of metastatic PCC/PGL that show accelerated growth after failure of current chemotherapies, and would be particularly interesting to test with MTT cells. In addition, cytostasis induced by pre-treatment with high concentrations of 5-aza might cause MPC and MTT cells to more closely resemble primary human PCC/PGL cultures, in which no tumor cells proliferate, thereby possibly increasing the relevance of both models.

Although our cytotoxicity results with human PCC/PGL cells provided proof of principle to justify further testing, cytotoxicity assays of these cells in primary cultures are challenging because the neoplastic cells can be rapidly overgrown by inevitably present fibroblasts and other cell types. The percentage of contaminating cell types varies greatly from tumor to tumor, probably reflecting variable histological characteristics of the tumor tissue [34]. The present study employed staining for a tumor cell specific marker, TH, to establish that tumor cells were being targeted. This methodology is well established but tedious, and future studies will require higher throughput methods.

Human PCC/PGL can be divided into clusters according to their gene expression profiles [35] that might in turn influence drug sensitivity. Tumors with *SDHB* mutations have a distinct pseudo-hypoxic signature. Because we were able to test only a small number of human tumors, we can not draw conclusions as to whether responsiveness to TOP inhibitors correlates with tumor genotype. However, a wide range of responsiveness among the three tested tumors from patients with *SDHB* mutations suggests that characteristics of individual tumors will be more important than genetic background.

A surprising finding in this study was increased expression of both luciferase and GFP in MPC GL-9 cells treated with camptothecin. This effect serves as an important reminder that chemotherapeutic agents can have unanticipated effects on gene expression. Interestingly, camptothecin has been reported to increase the expression of endogenous differentiation-related genes in cell lines derived from human hematopoietic tumors [36]. We therefore probed immunoblots for both luciferase and chromogranin A, a marker of neuroendocrine differentiation, but found that only luciferase and GFP were affected. Our experiments used a construct containing both luciferase and GFP driven by the CMV promoter. Since expression of both proteins is increased in parallel by camptothecin, the drug effect is most likely mediated by the CMV promoter.

Previous publications have called attention to increased CMV promoter-driven GFP expression in response to several drugs and other agents. Examples include 5-azacytidine [37], for which we also observed that effect, as well as histone deacetylase inhibitors, cisplatin and radiation [37], [38]. These agents act with different kinetics [38], suggesting a number of possible mechanisms. However, a likely explanation for our observations is drug-mediated demethylation of CpG motifs in the CMV promoter [39]. This can occur either in response to a methylation inhibitor such as 5-azacytidine [37] or to a histone deacetylase inhibitor, which would facilitate chromatin remodeling and removal of methylated DNA [40]. Topoisomerase inhibitors might produce a similar effect as DNA is unwound during transcription and, inversely, methylation can alter the number of cleavage sites produced in chromatin by topoisomerase inhibitors [41]. Transcriptional silencing of transgene expression by methylation has been demonstrated with a number of experimental models, and CpG-depleted DNA vectors have been tested as a tool to improve gene delivery systems [42]. Studies using luciferase constructs for that purpose have shown that CpG-containing reporter vectors are silenced by DNA methylation and that luminescence is increased by use of CpG-depleted vectors [43]. Because the CMV promoter, is widely utilized, it is important for investigators who use CMV-driven reporters for bioluminescence or fluorescence imaging studies to be aware of potential anomalous responses to TOP1 inhibitors or other drugs. In a preliminary in vivo experiment with subcutaneous MPC GL-9 cells we have found this to be a significant concern (JF Powers, unpublished data). A number of alternative approaches might be considered for optimal in vivo imaging and pre-clinical drug testing [44].

In summary, these results provide proof of principle for use of camptothecin or newer generation TOP1 inhibitors against PCC/PGL cells and suggest novel strategies for enhancing their efficacy and reducing their toxicity by optimizing both the combination and timing of their use in conjunction with other drugs. It should be borne in mind that TOP1 inhibitors and other drugs can cause anomalous increases in CMV reporter-controlled expression of luciferase and GFP, potentially confounding the interpretation of tumor imaging studies and pre-clinical drug testing.

## References

[1] DeLellis RA, Lloyd RV, Heitz PU, Eng C, editors (2004) Tumours of Endocrine Organs. Lyon: IARC Press.

[2] Pacak K, Eisenhofer G, Ahlman H, Bornstein SR, Gimenez-Roqueplo AP, et al (2007) Pheochromocytoma: recommendations for clinical practice from the First International Symposium. October 2005. Nat Clin Pract Endocrinol Metab 3: 92–102. 17237836 10.1038/ncpendmet0396

[3] Strong VE, Kennedy T, Al-Ahmadie H, Tang L, Coleman J, et al (2008) Prognostic indicators of malignancy in adrenal pheochromocytomas: clinical, histopathologic, and cell cycle/apoptosis gene expression analysis. Surgery 143: 759–768. 18549892 10.1016/j.surg.2008.02.007

[4] Tischler AS, Powers JF, Alroy J (2004) Animal models of pheochromocytoma. Histol Histopathol 19: 883–895. 15168351 10.14670/HH-19.883

[5] Ghayee HK, Bhagwandin VJ, Stastny V, Click A, Ding LH, et al (2013) Progenitor cell line (hPheo1) derived from a human pheochromocytoma tumor. PLoS One 8: e65624. 23785438 10.1371/journal.pone.0065624 PMC3681983

[6] Basili S, Moro S (2009) Novel camptothecin derivatives as topoisomerase I inhibitors. Expert Opin Ther Pat 19: 555–574. 19441934 10.1517/13543770902773437

[7] Nitiss JL (2009) DNA topoisomerase II and its growing repertoire of biological functions. Nat Rev Cancer 9: 327–337. 19377505 10.1038/nrc2608 PMC2730144

[8] Liu LF, Desai SD, Li TK, Mao Y, Sun M, et al (2000) Mechanism of action of camptothecin. Ann N Y Acad Sci 922: 1–10. 11193884 10.1111/j.1749-6632.2000.tb07020.x

[9] Park DS, Morris EJ, Greene LA, Geller HM (1997) G1/S cell cycle blockers and inhibitors of cyclin-dependent kinases suppress camptothecin-induced neuronal apoptosis. J Neurosci 17: 1256–1270. 9006970 10.1523/JNEUROSCI.17-04-01256.1997 PMC6793728

[10] Kenmotsu Y, Oshita F, Sugiura M, Murakami S, Kondo T, et al (2012) Nedaplatin and irinotecan in patients with large-cell neuroendocrine carcinoma of the lung. Anticancer Res 32: 1453–1456. 22493385

[11] Mairs RJ, Boyd M (2008) Optimizing MIBG therapy of neuroendocrine tumors: preclinical evidence of dose maximization and synergy. Nucl Med Biol 35 Suppl 1S9–20. 18707637 10.1016/j.nucmedbio.2008.04.008

[12] Gary-Bobo M, Hocine O, Brevet D, Maynadier M, Raehm L, et al (2012) Cancer therapy improvement with mesoporous silica nanoparticles combining targeting, drug delivery and PDT. Int J Pharm 423: 509–515. 22178618 10.1016/j.ijpharm.2011.11.045

[13] Chen KJ, Tang L, Garcia MA, Wang H, Lu H, et al (2012) The therapeutic efficacy of camptothecin-encapsulated supramolecular nanoparticles. Biomaterials 33: 1162–1169. 22074663 10.1016/j.biomaterials.2011.10.044 PMC3786683

[14] Ishiguro M, Iida S, Uetake H, Morita S, Makino H, et al (2007) Effect of combined therapy with low-dose 5-aza-2′-deoxycytidine and irinotecan on colon cancer cell line HCT-15. Ann Surg Oncol 14: 1752–1762. 17195906 10.1245/s10434-006-9285-4

[15] Yamagata Y, Szabo P, Szuts D, Bacquet C, Aranyi T, et al (2012) Rapid turnover of DNA methylation in human cells. Epigenetics 7: 141–145. 22395463 10.4161/epi.7.2.18906 PMC3335907

[16] Tischler AS, Ruzicka LA, Riseberg JC (1992) Immunocytochemical analysis of chromaffin cell proliferation in vitro. J Histochem Cytochem 40: 1043–1045. 1351491 10.1177/40.7.1351491

[17] Powers JF, Picard KL, Tischler AS (2009) RET expression and neuron-like differentiation of pheochromocytoma and normal chromaffin cells. Horm Metab Res 41: 710–714. 19551609 10.1055/s-0029-1224136

[18] Powers JF, Evinger MJ, Tsokas P, Bedri S, Alroy J, et al (2000) Pheochromocytoma cell lines from heterozygous neurofibromatosis knockout mice. Cell Tissue Res 302: 309–320. 11151443 10.1007/s004410000290

[19] Martiniova L, Perera SM, Brouwers FM, Alesci S, Abu-Asab M, et al (2011) Increased uptake of [(1)(2)(3)I]meta-iodobenzylguanidine, [(1)F]fluorodopamine, and [(3)H]norepinephrine in mouse pheochromocytoma cells and tumors after treatment with the histone deacetylase inhibitors. Endocr Relat Cancer 18: 143–157. 21098082 10.1677/ERC-10-0090 PMC4110720

[20] Martiniova L, Lai EW, Elkahloun AG, Abu-Asab M, Wickremasinghe A, et al (2009) Characterization of an animal model of aggressive metastatic pheochromocytoma linked to a specific gene signature. Clin Exp Metastasis 26: 239–250. 19169894 10.1007/s10585-009-9236-0 PMC3505859

[21] Korpershoek E, Pacak K, Martiniova L (2012) Murine models and cell lines for the investigation of pheochromocytoma: applications for future therapies? Endocr Pathol 23: 43–54. 22323007 10.1007/s12022-012-9194-y PMC3308007

[22] Nolting S, Grossman AB (2012) Signaling pathways in pheochromocytomas and paragangliomas: prospects for future therapies. Endocr Pathol 23: 21–33. 22391976 10.1007/s12022-012-9199-6

[23] Giubellino A, Woldemichael GM, Sourbier C, Lizak MJ, Powers JF, et al (2012) Characterization of two mouse models of metastatic pheochromocytoma using bioluminescence imaging. Cancer Lett 316: 46–52. 22154086 10.1016/j.canlet.2011.10.019 PMC3253957

[24] Jonsson E, Fridborg H, Csoka K, Dhar S, Sundstrom C, et al (1997) Cytotoxic activity of topotecan in human tumour cell lines and primary cultures of human tumour cells from patients. Br J Cancer 76: 211–219. 9231921 10.1038/bjc.1997.364 PMC2223940

[25] Kaufmann SH, Desnoyers S, Ottaviano Y, Davidson NE, Poirier GG (1993) Specific proteolytic cleavage of poly(ADP-ribose) polymerase: an early marker of chemotherapy-induced apoptosis. Cancer Res 53: 3976–3985. 8358726

[26] Tao K, Fang M, Alroy J, Sahagian GG (2008) Imagable 4T1 model for the study of late stage breast cancer. BMC Cancer 8: 228. 18691423 10.1186/1471-2407-8-228 PMC2529338

[27] Tone S, Sugimoto K, Tanda K, Suda T, Uehira K, et al (2007) Three distinct stages of apoptotic nuclear condensation revealed by time-lapse imaging, biochemical and electron microscopy analysis of cell-free apoptosis. Exp Cell Res 313: 3635–3644. 17643424 10.1016/j.yexcr.2007.06.018 PMC2705844

[28] Zhuang J, Ye Y, Liu X, Li F, Pan X, et al (2010) DNA demethylation in retinal neurocytes contributes to the upregulation of DNA repair protein, Ku80. Neuroreport 21: 282–286. 20145501 10.1097/WNR.0b013e328336ee7e

[29] Zacchetti D, Clementi E, Fasolato C, Lorenzon P, Zottini M, et al (1991) Intracellular Ca2+ pools in PC12 cells. A unique, rapidly exchanging pool is sensitive to both inositol 1,4,5-trisphosphate and caffeine-ryanodine. J Biol Chem 266: 20152–20158. 1657914

[30] Dobner PR, Tischler AS, Lee YC, Bloom SR, Donahue SR (1988) Lithium dramatically potentiates neurotensin/neuromedin N gene expression. J Biol Chem 263: 13983–13986. 2844750

[31] Kiss NB, Muth A, Andreasson A, Juhlin CC, Geli J, et al (2013) Acquired hypermethylation of the P16INK4A promoter in abdominal paraganglioma: relation to adverse tumor phenotype and predisposing mutation. Endocr Relat Cancer 20: 65–78. 23154831 10.1530/ERC-12-0267 PMC3573842

[32] Killian JK, Kim SY, Miettinen M, Smith C, Merino M, et al (2013) Succinate dehydrogenase mutation underlies global epigenomic divergence in gastrointestinal stromal tumor. Cancer Discov 3: 648–657. 23550148 10.1158/2159-8290.CD-13-0092 PMC4135374

[33] Milagre I, Nunes MJ, Moutinho M, Rivera I, Fuso A, et al (2010) Chromatin-modifying agents increase transcription of CYP46A1, a key player in brain cholesterol elimination. J Alzheimers Dis 22: 1209–1221. 20930312 10.3233/JAD-2010-100651

[34] Tischler AS (2008) Pheochromocytoma and extra-adrenal paraganglioma: updates. Arch Pathol Lab Med 132: 1272–1284. 18684026 10.5858/2008-132-1272-PAEPU

[35] Gimenez-Roqueplo AP, Dahia PL, Robledo M (2012) An update on the genetics of paraganglioma, pheochromocytoma, and associated hereditary syndromes. Horm Metab Res 44: 328–333. 22328163 10.1055/s-0031-1301302

[36] Aller P, Rius C, Mata F, Zorrilla A, Cabanas C, et al (1992) Camptothecin induces differentiation and stimulates the expression of differentiation-related genes in U-937 human promonocytic leukemia cells. Cancer Res 52: 1245–1251. 1737386

[37] Kamensek U, Sersa G, Vidic S, Tevz G, Kranjc S, et al (2011) Irradiation, cisplatin, and 5-azacytidine upregulate cytomegalovirus promoter in tumors and muscles: implementation of non-invasive fluorescence imaging. Mol Imaging Biol 13: 43–52. 20396957 10.1007/s11307-010-0300-6 PMC3023030

[38] Grassi G, Maccaroni P, Meyer R, Kaiser H, D'Ambrosio E, et al (2003) Inhibitors of DNA methylation and histone deacetylation activate cytomegalovirus promoter-controlled reporter gene expression in human glioblastoma cell line U87. Carcinogenesis 24: 1625–1635. 12869421 10.1093/carcin/bgg118

[39] Brooks AR, Harkins RN, Wang P, Qian HS, Liu P, et al (2004) Transcriptional silencing is associated with extensive methylation of the CMV promoter following adenoviral gene delivery to muscle. J Gene Med 6: 395–404. 15079814 10.1002/jgm.516

[40] Jones PL, Veenstra GJ, Wade PA, Vermaak D, Kass SU, et al (1998) Methylated DNA and MeCP2 recruit histone deacetylase to repress transcription. Nat Genet 19: 187–191. 9620779 10.1038/561

[41] Leteurtre F, Kohlhagen G, Fesen MR, Tanizawa A, Kohn KW, et al (1994) Effects of DNA methylation on topoisomerase I and II cleavage activities. J Biol Chem 269: 7893–7900. 8132507

[42] Yew NS, Zhao H, Przybylska M, Wu IH, Tousignant JD, et al (2002) CpG-depleted plasmid DNA vectors with enhanced safety and long-term gene expression in vivo. Mol Ther 5: 731–738. 12027557 10.1006/mthe.2002.0598

[43] Klug M, Rehli M (2006) Functional analysis of promoter CpG methylation using a CpG-free luciferase reporter vector. Epigenetics 1: 127–130. 17965610 10.4161/epi.1.3.3327

[44] Gu L, Hall DJ, Qin Z, Anglin E, Joo J, et al (2013) In vivo time-gated fluorescence imaging with biodegradable luminescent porous silicon nanoparticles. Nat Commun 4: 2326. 23933660 10.1038/ncomms3326 PMC4154512

