# Correction: Cytocidal Activities of Topoisomerase 1 Inhibitors and 5-Azacytidine against Pheochromocytoma/Paraganglioma Cells in Primary Human Tumor Cultures and Mouse Cell Lines

**DOI:** 10.1371/journal.pone.0092492

**Published:** 2014-03-10

**Authors:** The PLOS ONE Staff

The Citation is incorrect. The correct Citation is: Powers JF, Korgaonkar PG, Fliedner S, Giubellino A, Pacak K, et al. (2014) Cytocidal Activities of Topoisomerase 1 Inhibitors and 5-Azacytidine against Pheochromocytoma/Paraganglioma Cells in Primary Human Tumor Cultures and Mouse Cell Lines. PLoS ONE 9(2): e87807. doi:10.1371/journal.pone.0087807
